# Diagnostic accuracy of peripheral venous lactate and the 2009 WHO warning signs for identifying severe dengue in Thai adults: a prospective observational study

**DOI:** 10.1186/s12879-016-1386-5

**Published:** 2016-02-01

**Authors:** Vipa Thanachartwet, Anan Wattanathum, Nittha Oer-areemitr, Akanitt Jittmittraphap, Duangjai Sahassananda, Chalida Monpassorn, Manoon Surabotsophon, Varunee Desakorn

**Affiliations:** 1 Department of Clinical Tropical Medicine, Faculty of Tropical Medicine, Mahidol University, Bangkok, 10400 Thailand; 2 Pulmonary and Critical Care Division, Department of Medicine, Phramongkutklao Hospital, 315 Rajvithi Road, Ratchathewi District, Bangkok, 10400 Thailand; 3 Department of Microbiology and Immunology, Faculty of Tropical Medicine, Mahidol University, Bangkok, 10400 Thailand; 4 Information Technology Unit, Faculty of Tropical Medicine, Mahidol University, Bangkok, 10400 Thailand; 5 Hospital for Tropical Diseases, Faculty of Tropical Medicine, Mahidol University, Bangkok, 10400 Thailand; 6 Pulmonary and Critical Care Division, Department of Medicine, Ramkhamhaeng Hospital, Bangkok, 10240 Thailand

**Keywords:** Severe dengue, Lactate, Warning signs, Diagnostic accuracy, Prospective study

## Abstract

**Background:**

Dengue is the most common mosquito-borne viral disease in humans. However, the sensitivities of warning signs (WSs) for identifying severe dengue in adults are low, and the utility of lactate levels for identifying severe dengue in adults has not been verified. Therefore, we aimed to evaluate the diagnostic accuracy of using peripheral venous lactate levels (PVL), as well as WSs established by the World Health Organization, for identifying severe dengue.

**Methods:**

We prospectively evaluated individuals hospitalized for dengue who were admitted to the Hospital for Tropical Diseases in Thailand between May 2013 and January 2015. Blood samples to evaluate PVL levels were collected at admission and every 24 h until the patient exhibited a body temperature of <37.8 °C for at least 24 h. Data were recorded on a pre-defined case report form, including baseline characteristics, clinical parameters, and laboratory findings.

**Results:**

Among 125 patients with confirmed dengue, 105 (84.0 %) patients had non-severe dengue, and 20 (16.0 %) patients had severe dengue. The presence of clinical fluid accumulation as a WS provided high sensitivity (75.0 %, 95 % confidence interval [CI]: 50.9–91.3 %) and specificity (90.5 %, 95 % CI: 83.2–95.3 %). The PVL level at admission was used to evaluate its diagnostic value, and receiver operating characteristic curve analysis revealed an area under the curve of 0.84 for identifying severe dengue. At the optimal cutoff value (PVL: 2.5 mmol/L), the sensitivity and specificity were 65.0 % (95 % CI: 40.8–84.6 %) and 96.2 % (95 % CI: 90.5–99.0 %), respectively. A combined biomarker comprising clinical fluid accumulation and/or PVL of ≥2.5 mmol/L provided the maximum diagnostic accuracy for identifying severe dengue, with a sensitivity of 90.0 % (95 % CI: 68.3–98.8 %) and a specificity of 87.6 % (95 % CI: 79.8–93.2 %).

**Conclusions:**

Clinical fluid accumulation and/or PVL may be used as a diagnostic biomarker of severe dengue among adults. This biomarker may facilitate early recognition and timely treatment of patients with severe dengue, which may reduce dengue-related mortality and hospital burden.

## Background

Dengue is the most common global mosquito-borne viral disease in humans [1]. Dengue is currently endemic in more than 125 countries, and Central and South America, South-East Asia, and the Western Pacific region are the most seriously affected [2, 3]. In their 2020 goals for dengue, the World Health Organization (WHO) called for a 25 % reduction in morbidity and a 50 % reduction in mortality [1]. The key to reducing mortality from severe dengue is early recognition and timely treatment before the patient develops dengue shock and organ dysfunction [3].

Previous reports have demonstrated that there has been an epidemic shift from children to adults regarding those who experience more severe dengue [2, 4, 5]. In Thailand, a previous study reported that the number of adults with dengue has increased dramatically during recent decades [4]; more so, approximately 27.9 % of all hospitalized adults with dengue develop severe dengue, according to the 2009 WHO definitions [6]. A recent systematic review regarding the application of the 2009 WHO warning signs (WSs) for identifying severe dengue revealed a wide range of sensitivities (59–98 %) and specificities (41–99 %) [7]. In adults with dengue, the WSs have low sensitivities for identifying severe dengue, as they were developed based on findings that were limited by retrospective designs and small sample sizes [8, 9]. Unfortunately, using WSs to identify severe dengue increases the hospital burden and work load of healthcare personnel as much as two fold. This is especially true for resource-limited countries and areas with endemic dengue, particularly Thailand [10, 11].

Following dengue virus infection, the complications of dengue may be caused by an immunologic mechanism that results in increased capillary permeability, which leads to altered microcirculatory blood flow and ultimately to decreased tissue perfusion during the critical phase of dengue [12]. Under anaerobic conditions or in tissues with poor perfusion, lactate is formed by the reduction of pyruvate via the enzyme lactate dehydrogenase in order to produce ATP for cellular energy. In this context, serum lactate has been demonstrated to be a prognostic marker in critically ill patients [13, 14]. Several reports have also demonstrated that serum lactate in critically ill patients was correlated with in-hospital mortality and organ dysfunction [15–21]. In a previous study, we showed that serum lactate is an independent factor for identifying severe dengue in adults [22]. Thus, we hypothesized that elevated lactate levels might serve as a diagnostic biomarker for identifying tissue hypoperfusion among patients with dengue before clinical manifestations of severe dengue develop. To investigate this hypothesis, we performed this prospective observational study among patients with dengue who were admitted to the Hospital for Tropical Diseases in Bangkok, Thailand. Using data from these patients, we evaluated the diagnostic accuracy of peripheral venous lactate (PVL) and WSs for identifying severe dengue.

## Methods

### Ethical considerations

The study’s design was approved by the Ethics Committee of the Faculty of Tropical Medicine, Mahidol University (MUTM 2013-008-02) and the Ethics Committee of Phramongkutklao Hospital, Bangkok, Thailand (IRBRTA 1571/2013). The Standards for Reporting of Diagnostic Accuracy (STARD) were followed in this study [23]. Prior to participating in the study, written informed consent was obtained from all patients, (and/or the patient's guardians if the patient was 15–18 years old).

### Study design

This prospective observational study was performed among patients who were admitted to the Hospital for Tropical Diseases (Faculty of Tropical Medicine, Mahidol University, Bangkok, Thailand) between May 2013 and January 2015. Individuals ≥15 years old were eligible for inclusion if they presented with clinical indicators of dengue, which were defined as acute fever and ≥2 of the following symptoms: 1) headache, 2) retro-orbital pain or ocular pain, 3) myalgia, 4) arthralgia, 5) rash, 6) a positive tourniquet test (≥20 petechiae per 1 square inch), or 7) leukopenia (defined as a white blood cell count <5.0 × 10^3^ cells/μL). In addition, to be eligible for inclusion, participants had to have the presence of dengue viral infection confirmed via reverse-transcriptase polymerase chain reaction (RT-PCR) from an admission serum sample or dengue-specific immunoglobulin M (IgM) and immunoglobulin G (IgG) using enzyme-linked immunosorbent assays (ELISA) of paired serum samples (taken upon admission and 2 weeks after admission). Patients with a history of underlying medical illness, mixed infection, or who were currently pregnant were excluded from this study.

Laboratory tests at admission included a complete blood count, blood chemistry analysis, and assessment of PVL levels. Blood samples for PVL were subsequently collected every 24 h until the patient exhibited a body temperature of <37.8 °C for at least 24 h. To exclude the presence of other infections, hospitalized patients provided two blood cultures for microbiology analysis and underwent a urinalysis and chest radiography at admission. Other diagnostic tests for infectious diseases were also performed when clinical findings were suspect. The treating physicians were blinded to the results of the PVL tests. All patients with dengue received standard treatment according to the 2009 WHO guidelines for dengue [24]. All patient data, including baseline characteristics, clinical parameters, and laboratory findings, were recorded on a pre-defined case report form.

### Case definitions for dengue

Using the 2009 WHO dengue case definitions [24], we classified patients as having either non-severe or severe dengue on the basis of their clinical and laboratory data. Patients with non-severe dengue were sub-categorized according to the presence or absence of WSs. Non-severe dengue without WSs was defined as an acute fever with ≥2 of the following symptoms: 1) nausea, 2) vomiting, 3) rash, 4) myalgia, 5) arthralgia, 6) a positive tourniquet test, or 7) leukopenia. The WSs included 1) abdominal pain; 2) persistent vomiting (defined as vomiting with signs of dehydration during physical examination); 3) clinical fluid accumulation that manifested as pleural effusion, ascites, or a serum albumin level <3.5 g/dL; 4) lethargy; 5) a liver span of >15 cm; 6) bleeding from a mucosal area, including the nose, gums, gastrointestinal tract, or vagina; 7) an increase in hematocrit of 2 % above the sex-specific reference range for a healthy Thai adult; and 8) a platelet count of ≤100 × 10^3^/μL. Patients were diagnosed with severe dengue if they met all of the criteria for non-severe dengue, as well as at least one of the following: 1) severe plasma leakage that caused shock or respiratory distress, 2) severe clinical bleeding (i.e., bleeding in the vital organs or spontaneous bleeding from a mucosal area that required a blood transfusion), or 3) severe organ involvement (evidenced by an aspartate aminotransferase [AST] level >1,000 U/L, an alanine aminotransferase [ALT] level >1,000 U/L, a serum creatinine level ≥3 times above baseline, myocarditis, and/or encephalitis). Plasma leakage was defined as a ≥20 % increase in hematocrit (above the reference range) or clinical evidence of fluid accumulation. Shock was defined as a systolic blood pressure of <90 mmHg with a <0.5 mL/kg/h decrease in urine output, impaired consciousness, AST level >1,000 IU/L, or ALT level >1,000 IU/L. Respiratory distress was defined as a respiratory rate of ≥24 breaths/min with <95 % oxygen saturation on room air and/or the need for oxygen therapy.

### RT-PCR

Dengue viral RNA was extracted from each patient’s serum using a 2-step PCR method, as described by Lanciotti et al*.* [25] and modified using the methods outlined by Reynes et al. [26]. The RNA was extracted from the acute serum samples using the PureLink® Viral RNA/DNA Mini Kit (Invitrogen™, USA), according to the manufacturer’s instructions.

### Serology for dengue viral infection

All sera were tested using 4 separate captures in ELISA assays for IgM and IgG against the dengue virus and the Japanese encephalitis virus, as described by Innis et al. [27]. To differentiate between dengue and other flavivirus infections, we determined the ratio of dengue IgM to Japanese encephalitis virus IgM, with a ratio of ≥1.0 indicating dengue virus infection and a ratio of <1.0 indicating other flavivirus infection. The ratio of dengue IgM to dengue IgG was also calculated, with a ratio of ≥1.8 indicating a primary dengue infection and a ratio of <1.8 indicating a secondary dengue infection.

### Measurement of peripheral venous lactate

Blood samples were collected from a vein in an upper extremity without the use of a tourniquet. The 2 mL blood sample was placed in a vacutainer that contained sodium fluoride, and the sample was immediately placed on ice, sent to the laboratory center, and analyzed for lactate within 10 min of being drawn. A colorimetric assay with an enzymatic reaction that converted lactate to pyruvate was performed using an auto-analyzer (Roche/Hitachi Cobas C Systems, USA), according to the manufacturers' protocols. In this assay, l-lactate is oxidized to pyruvate and hydrogen peroxide by lactate oxidase, and the hydrogen peroxide reacts with peroxidase to generate a colored dye. The intensity of the color is directly proportional to the l-lactate concentration, as measured by the increase in absorbance. Lactate levels are expressed as mmol/L. The laboratory personnel were blinded to the sample sources, and the coefficient of variation for this assay in our laboratory was 1.1 %.

### Sample size calculation

A previous retrospective study at the Hospital for Tropical Diseases indicated that the incidence of severe dengue was 27.9 % among adults who were hospitalized with dengue according to the 2009 WHO definitions [6]. However, it has also been estimated that the incidence of severe dengue would be approximately 20 % among dengue patients if they received close observation throughout treatment and care during their hospitalization [22]. Based on this information, we calculated that a sample size of 120 patients was needed for this study, using a specificity of 90 % with a confidence interval of ±6 %.

### Statistical analysis

All data were analyzed using SPSS software (version 18.0; SPSS Inc., Chicago, IL). Numerical variables were tested for normality using the Kolmogorov-Smirnov test. Variables with non-normal distribution were summarized as a median and inter-quartile range (IQR) and were compared using the Mann–Whitney *U* test for 2-group comparison. Categorical variables are expressed as frequencies and percentages, and were analyzed using the Chi-square test or Fisher’s exact test, as appropriate. The optimal lactate cutoff value for identifying severe dengue was determined using receiver operating characteristic (ROC) curves. Diagnostic parameters were evaluated using 2 × 2 tables and 95 % confidence intervals (CI) to determine their sensitivity, specificity, negative predictive value (NPV), positive predictive value (PPV), positive likelihood ratio (LR+), and negative likelihood ratio (LR–). All tests of significance were 2-sided, with a *p*-value <0.05 indicating statistical significance.

## Results

### Patient characteristics

**Fig. 1:**
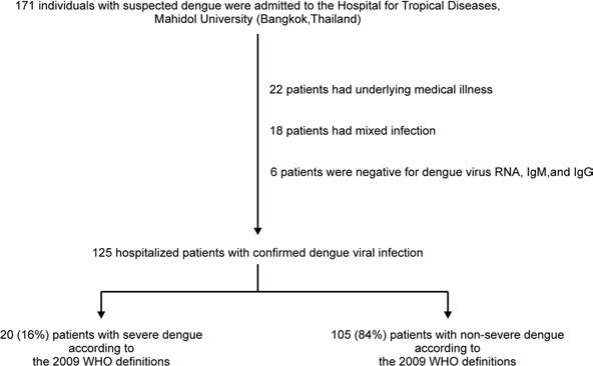
Study flow chart. *WHO* World Health Organization

Among the 171 individuals with suspected dengue who were admitted to the Hospital for Tropical Diseases between May 2013 and January 2015, 46 patients were excluded for various causes, including a history of underlying illness (22 patients, 47.8 %), the presence of mixed infection (18 patients, 39.1 %), or negative RT-PCR/ELISA results for dengue (6 patients, 13.1 %). Thus, 125 hospitalized patients with confirmed dengue viral infection were included in this study. Among these 125 patients, 105 (84.0 %) patients had non-severe dengue, including 5 (4.8 %) patients without WSs and 100 (95.2 %) patients with WSs. Twenty (16.0 %) patients had severe dengue according to the 2009 WHO definitions, including 15 (75.0 %) patients with severe plasma leakage, 12 (80.0 %) patients with plasma leakage and shock, and 8 (53.3 %) patients with plasma leakage and respiratory distress. Among the 20 patients with severe dengue, 11 (55.0 %) patients had severe clinical bleeding. Eight (40.0 %) patients had severe organ involvement, including 5 (62.5 %) patients with an AST level >1,000 U/L and/or an ALT level >1,000 U/L, 4 (50.0 %) patients with a serum creatinine level ≥3 times above the baseline, 2 (25.0 %) patients with myocarditis, and 1 (12.5 %) patient with encephalitis (Fig. 1).

### Comparison of clinical and laboratory characteristics of patients with severe and non-severe dengue

At admission, patients with severe and non-severe dengue had similar clinical and laboratory characteristics. However, patients with severe dengue were significantly more likely to be older (median [IQR]: 36.5 [20.2–50.5] years vs. 23.0 [19.0–33.5] years, *p* = 0.010). In addition, patients diagnosed with severe dengue were more likely to present with a longer duration of fever (median [IQR]: 5.0 [4.0–6.0] days vs. 4.0 [3.0–5.0] days, *p* = 0.003), have persistent vomiting (8/20 [40.0 %] patients vs. 12/105 [11.4 %] patients, *p* = 0.004), and have an increased respiratory rate (median [IQR]: 22 [20–24] breaths/min vs. 20 [18–22] breaths/min, *p* = 0.019). These patients were also more likely to have mucosal bleeding (14/20 [70.0 %] patients vs. 39/105 [37.1 %] patients, *p* = 0.013), skin bleeding (13/20 [65.0 %] patients vs. 22/105 [21.0 %], *p* < 0.001), a liver span of >15 cm (12/20 [60.0 %] patients vs. 17/105 [16.2 %] patients, *p* < 0.001], and clinical fluid accumulation (15/20 [75.0 %] patients vs. 10/105 [9.5 %] patients, *p* < 0.001). In addition, patients with severe dengue had significantly higher levels of AST (median [IQR]: 228 [162–760] IU/L vs. 71 [36–156] IU/L, *p* < 0.001), ALT (median [IQR]: 172 [114–415] IU/L vs. 46 [18–102] IU/L, *p* < 0.001), and lactate (median [IQR]: 2.7 [1.8–3.1] mmol/L vs. 1.4 [1.2–1.8] mmol/L, *p* < 0.001). Furthermore, patients with severe dengue had significantly lower platelet counts (median [IQR]: 41.5 [11.2–63.5] × 10^3^/μL vs. 90.0 [56.0–145.5] × 10^3^/μL, *p* < 0.001] and albumin levels (median [IQR]: 3.6 [3.1–4.2] g/dL vs. 4.3 [4.0–4.5] g/dL, *p* < 0.001). Moreover, patients with severe dengue had a significantly longer hospitalization duration compared to patients with non-severe dengue (median [IQR]: 4.8 [2.9–9.8] days vs. 3.7 [2.7–4.9] days, *p* = 0.012) (Table 1).

**Table 1:** Baseline clinical and laboratory characteristics of 125 patients hospitalized with dengue

Characteristics	All dengue cases		The 2009 WHO dengue case definitions				*p*-value
			Severe dengue		Non-severe dengue		
	n	No. (%)	n	No. (%)	n	No. (%)	
Baseline characteristics							
Age (years), median (IQR)	125	24.0 (19.0–36.0)	20	36.5 (20.2–50.5)	105	23.0 (19.0–33.5)	0.010^c^
Sex, female	125	57 (45.6)	20	11 (55.0)	105	46 (43.8)	0.499^a^
Residential area, Bangkok	125	104 (83.2)	20	18 (90.0)	105	86 (81.9)	0.523^b^
Clinical symptoms							
Fever (days), median (IQR)	125	4.0 (3.0–5.0)	20	5.0 (4.0–6.0)	105	4.0 (3.0–5.0)	0.003^c^
Myalgia	125	114 (91.2)	20	19 (95.0)	105	95 (90.5)	1.000^b^
Headache	125	109 (87.2)	20	17 (85.0)	105	92 (87.6)	0.720^b^
Lethargy	125	94 (75.2)	20	18 (90.0)	105	76 (72.4)	0.155^b^
Nausea	125	88 (70.4)	20	14 (70.0)	105	74 (70.5)	1.000^a^
Retro-orbital pain	125	80 (64.0)	20	11 (55.0)	105	69 (65.7)	0.509^a^
Abdominal pain	125	53 (42.4)	20	9 (45.0)	105	44 (41.9)	0.992^a^
Arthralgia	125	41 (32.8)	20	9 (45.0)	105	32 (30.5)	0.313^a^
Persistent vomiting	125	20 (16.0)	20	8 (40.0)	105	12 (11.4)	0.004^b^
Physical examinations							
Temperature (°C), median (IQR)	125	38.5 (37.8–39.2)	20	38.5 (37.7–39.0)	105	38.5 (37.8–39.2)	0.580^c^
Mean arterial pressure (mmHg), median (IQR)	125	86 (78–92)	20	84 (70–93)	105	86 (78–92)	0.522^c^
Heart rate (beats/min), median (IQR)	125	80 (66–90)	20	83 (70–91)	105	79 (66–90)	0.457^c^
Respiratory rate (breaths/min), median (IQR)	125	22 (20–22)	20	22 (20–24)	105	20 (18–22)	0.019^c^
Tourniquet test, positive	125	106 (84.8)	20	15 (75.0)	105	91 (86.7)	0.187^b^
Rash	125	55 (44.0)	20	9 (45.0)	105	46 (43.8)	1.000^a^
Mucosal bleeding	125	53 (42.4)	20	14 (70.0)	105	39 (37.1)	0.013^a^
Skin bleeding	125	35 (28.0)	20	13 (65.0)	105	22 (21.0)	<0.001^a^
Liver span of >15 cm	125	29 (23.2)	20	12 (60.0)	105	17 (16.2)	<0.001^b^
Clinical fluid accumulation	125	25 (20.0)	20	15 (75.0)	105	10 (9.5)	<0.001^b^
Confirmation tests for dengue							
Dengue RT-PCR, positive	125	79 (63.2)	20	15 (75.0)	105	64 (61.0)	1.000^b^
Serotypes 1 or 4	79	22 (27.8)	15	4 (26.7)	64	18 (28.1)	
Serotypes 2 or 3	79	57 (72.2)	15	11 (73.3)	64	46 (71.9)	
Dengue ELISA, positive	125	119 (95.2)	20	20 (100)	105	99 (94.3)	0.526^b^
Primary dengue infection	119	4 (3.4)	20	1 (5.0)	99	3 (3.0)	
Secondary dengue infection	119	115 (96.6)	20	19 (95.0)	99	96 (97.0)	
Complete blood counts							
Hemoglobin, median (IQR), g/dL	125	13.8 (12.8–14.9)	20	13.9 (12.7–16.7)	105	13.8 (12.8–14.9)	0.540^c^
Hematocrit, median (IQR), %	125	41.6 (38.1–44.4)	20	40.7 (37.1–48.9)	105	41.6 (38.4–44.4)	0.882^c^
WBC, median (IQR), ×10^3^/μL	125	3.0 (2.2–4.8)	20	3.6 (2.1–6.8)	105	3.0 (2.2–4.6)	0.324^c^
Platelet counts, median (IQR), ×10^3^/μL	125	78.0 (48.0–127.0)	20	41.5 (11.2–63.5)	105	90.0 (56.0–145.5)	<0.001^c^
Blood biochemistry							
Creatinine, median (IQR), mg/dL	125	0.8 (0.6–1.0)	20	0.8 (0.6–1.1)	105	0.9 (0.7–1.0)	0.859^c^
Albumin, median (IQR), g/dL	125	4.2 (3.9–4.5)	20	3.6 (3.1–4.2)	105	4.3 (4.0–4.5)	<0.001^c^
AST, median (IQR), IU/L	125	94 (38–202)	20	228 (162–760)	105	71 (36–156)	<0.001^c^
ALT, median (IQR), IU/L	125	65 (22–148)	20	172 (114–415)	105	46 (18–102)	<0.001^c^
Lactate level, median (IQR), mmol/L	125	1.5 (1.2–1.9)	20	2.7 (1.8–3.1)	105	1.4 (1.2–1.8)	<0.001^c^
Duration of hospitalization (days), median (IQR)	125	3.9 (2.8–5.0)	20	4.8 (2.9–9.8)	105	3.7 (2.7–4.9)	0.012^c^

^a^Chi-square test; ^b^Fisher’s exact test; ^c^Mann-Whitney *U* test; *WHO* World Health Organization; *IQR* interquartile range; *RT-PCR* reverse-transcriptase polymerase chain reaction; *ELISA* enzyme-linked immunosorbent assay; *WBC* white blood cell count; *AST* aspartate aminotransferase; *ALT* alanine aminotransferase

### Diagnostic value of the 2009 WHO warning signs for identifying severe dengue

**Fig. 2:**
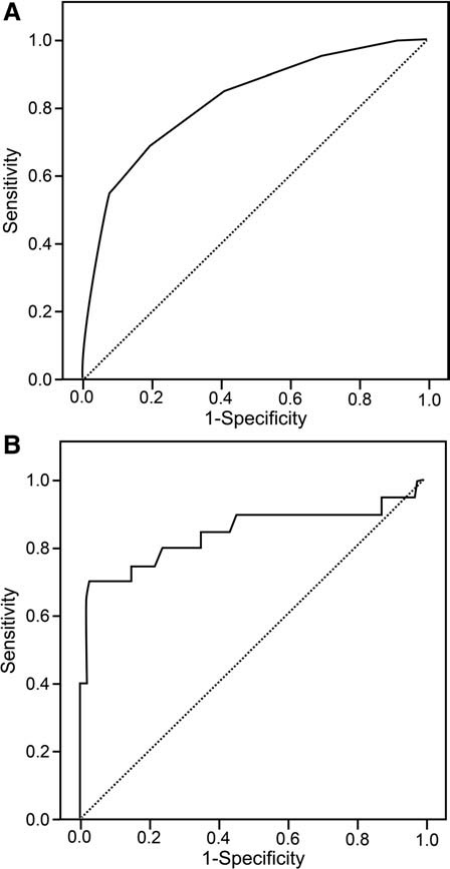
ROC curves for 2009 WHO warning signs and peripheral venous lactate in identifying severe dengue. **a** The AUROC for the number of 2009 WHO warning signs at admission was 0.82 (95 % confidence interval [95 % CI]: 0.72–0.93). **b** The AUROC for peripheral venous lactate at admission was 0.84 (95 % CI: 0.72–0.97). *AUROC* area under the receiver operating characteristics; *ROC* receiver operating characteristics; *WHO* World Health Organization

The diagnostic values of the 2009 WHO WSs for identifying severe dengue at admission are shown in Table 2. The sensitivities for all WSs were low, except for lethargy (90.0 %, 95 % CI: 68.3–98.8 %), clinical fluid accumulation (75.0 %, 95 % CI: 50.9–91.3 %), and mucosal bleeding (70.0 %, 95 % CI: 45.7–88.1 %). Similarly, the specificities for all WSs were low, except for persistent vomiting (88.6 %, 95 % CI: 80.9–94.0 %), clinical fluid accumulation (90.5 %, 95 % CI: 83.2–95.3 %), and liver span of >15 cm (83.8 %, 95 % CI: 75.4–90.3 %). The PPVs and LR+ values for the WSs were low, although the NPVs for the WSs were high. The LR– values for the WSs ranged from 0.3 to 0.9, which indicated a minimal to slightly decreased chance of developing severe dengue. However, the area under the ROC (AUROC) for the number of WSs in identifying severe dengue was 0.82 (95 % CI: 0.72–0.93) (Fig. 2a). The optimal sensitivity (70.0 %, 95 % CI: 45.7–88.1 %) and specificity (80.0 %, 95 % CI: 71.1–87.2 %) were obtained using ≥4 WSs, which provided a low PPV (40.0 %, 95 % CI: 23.9–57.9 %) and a high NPV (93.3 %, 95 % CI: 86.0–97.5 %), as well as a LR+ of 3.5 (95 % CI: 2.2–5.6) and a LR– of 0.4 (95 % CI: 0.2–0.7). Therefore, clinical fluid accumulation as a WS demonstrated good diagnostic value for identifying severe dengue among patients hospitalized with dengue.

**Table 2:** Diagnostic value of warning signs at admission according to dengue severity

	The 2009 WHO definitions		Sensitivity (95 % CI)	Specificity (95 % CI)	PPV (95 % CI)	NPV (95 % CI)	LR+ (95 % CI)	LR– (95 % CI)
	Severe (n = 20)	Non-severe (n = 105)						
Individual warning sign								
Abdominal pain	9	44	45.0 (23.1–68.5)	58.1 (48.1–67.7)	17.0 (8.1–29.8)	84.7 (74.3–92.1)	1.1 (0.6–1.8)	0.9 (0.6–1.4)
Persistent vomiting	8	12	40.0 (19.1–64.0)	88.6 (80.9–94.0)	40.0 (19.1–64.0)	88.6 (80.9–94.0)	3.5 (1.6–7.4)	0.7 (0.5–1.0)
Clinical fluid accumulation	15	10	75.0 (50.9–91.3)	90.5 (83.2–95.3)	60.0 (38.7–78.9)	95.0 (88.7–98.4)	7.9 (4.2–15.0)	0.3 (0.1–0.6)
Lethargy	18	76	90.0 (68.3–98.8)	27.6 (19.3–37.2)	19.2 (11.8–28.6)	93.6 (78.6–99.2)	1.2 (1.0–1.5)	0.4 (0.1–1.4)
Liver span of >15 cm	12	17	60.0 (36.0–80.9)	83.8 (75.4–90.3)	41.4 (23.5–61.1)	91.7 (84.2–96.3)	3.7 (2.1–6.5)	0.5 (0.3–0.8)
Mucosal bleeding	14	39	70.0 (45.7–88.1)	62.9 (52.9–72.1)	26.4 (15.3–40.3)	91.7 (82.7–96.9)	1.9 (1.3–2.8)	0.5 (0.2–1.0)
Hematocrit >2 % and platelets ≤100 × 10^3^/μL	12	42	60.0 (36.0–80.9)	60.0 (50.0–69.4)	22.2 (12.0–35.6)	88.7 (79.0–95.0)	1.5 (1.0–2.3)	0.7 (0.4–1.2)
Number of warning signs								
≥ 1	20	95	100.0 (83.2–100.0)	9.5 (4.7–16.8)	17.4 (11.0–25.6)	100.0 (69.2–100.0)	1.1 (1.0–1.2)	0
≥ 2	19	71	95.0 (75.1–99.9)	32.4 (23.6–42.1)	21.1 (13.2–31.0)	97.1 (85.1–99.9)	1.4 (1.2–1.7)	0.2 (0–1.1)
≥ 3	17	43	85.0 (62.1–96.8)	59.0 (49.0–68.6)	28.3 (17.4–41.4)	95.4 (87.1–99.0)	2.1 (1.6–2.8)	0.2 (0.1–0.7)
≥ 4	14	21	70.0 (45.7–88.1)	80.0 (71.1–87.2)	40.0 (23.9–57.9)	93.3 (86.0–97.5)	3.5 (2.2–5.6)	0.4 (0.2–0.7)
≥ 5	11	8	55.0 (31.5–76.9)	92.4 (85.5–96.6)	57.9 (33.5–79.8)	91.5 (84.5–96.0)	7.2 (3.3–15.7)	0.5 (0.3–0.8)
≥ 6	5	2	25.0 (8.7–49.1)	98.1 (93.3–99.8)	71.4 (29.0–96.3)	87.3 (79.9–92.7)	13.1 (2.7–63.0)	0.8 (0.6–1.0)

*WHO* World Health Organization; *CI* confidence interval; *PPV* positive predictive value; *NPV* negative predictive value; *LR+* positive likelihood ratio; *LR–* negative likelihood ratio

### Diagnostic value of PVL for identifying severe dengue

We initially evaluated the diagnostic value of PVL for identifying severe dengue at admission. The AUROC for PVL in identifying severe dengue was 0.84 (95 % CI: 0.72–0.97) (Fig. 2b). At the optimal PVL cutoff value of 2.5 mmol/L, the sensitivity was 65.0 % (95 % CI: 40.8–84.6 %), the specificity was 96.2 % (95 % CI: 90.5–99.0 %), the PPV was 76.5 % (95 % CI: 50.1–93.2 %), the NPV was 93.5 % (95 % CI: 87.1–97.4 %), the LR+ was 17.1 (95 % CI: 6.2–47.0), and the LR– was 0.4 (95 % CI: 0.2–0.7) (Table 3). To improve the diagnostic accuracy for identifying severe dengue, we developed a combined biomarker consisting of clinical fluid accumulation and/or PVL ≥2.5 mmol/L, which revealed improvements in sensitivity (90.0 %, 95 % CI: 68.3–98.8 %), specificity (87.6 %, 95 % CI: 79.8–93.2 %), PPV (58.1 %, 95 % CI: 39.1–75.4 %), NPV (97.9 %, 95 % CI: 92.5–99.7 %), LR+ (7.3, 95 % CI: 4.3–12.3), and LR– (0.1, 95 % CI: 0–0.4) (Table 3).

**Table 3:** Diagnostic value of lactate at admission and a combination of clinical fluid accumulation with lactate at admission

Cutoff value	The 2009 WHO definitions		Sensitivity (95 % CI)	Specificity (95 % CI)	PPV (95 % CI)	NPV (95 % CI)	LR+ (95 % CI)	LR– (95 % CI)
	Severe (n = 20)	Non-severe (n = 105)						
Lactate at admission (mmol/L)								
≥ 1.0 mmol/L	19	96	95.0 (75.1–99.9)	8.6 (4.0–15.6)	16.5 (10.2–24.6)	90.0 (55.5–99.8)	1.0 (0.9–1.2)	0.6 (0.1–4.4)
≥ 1.5 mmol/L	18	51	90.0 (68.3–98.8)	51.4 (41.5–61.3)	26.1 (16.2–38.1)	96.4 (87.7–99.6)	1.8 (1.4–2.4)	0.2 (0.1–0.7)
≥ 2.0 mmol/L	14	15	70.0 (45.7–88.1)	85.7 (77.5– 91.8)	48.3 (29.4–67.47)	93.8 (86.9–97.7)	4.9 (2.8–8.5)	0.4 (0.2–0.7)
≥ 2.5 mmol/L	13	4	65.0 (40.8–84.6)	96.2 (90.5–99.0)	76.5 (50.1–93.2)	93.5 (87.1–97.4)	17.1 (6.2–47.0)	0.4 (0.2–0.7)
Clinical fluid accumulation and/or lactate at admission (mmol/L)								
≥ 1.0 mmol/L	20	96	100.0 (83.2–100.0)	8.6 (4.0-15.6)	17.2 (10.9–25.4)	100.0 (66.4–100.0)	1.1 (1.0–1.2)	0
≥ 1.5 mmol/L	20	55	100.0 (83.2–100.0)	47.6 (37.8–57.6)	26.7 (17.1–38.1)	100.0 (92.9–100.0)	1.9 (1.6–2.3)	0
≥ 2.0 mmol/L	18	22	90.0 (68.3–98.8)	79.1 (70.0–86.4)	45.0 (29.3–61.5)	97.6 (91.8–99.7)	4.3 (2.9–6.4)	0.1 (0–0.5)
≥ 2.5 mmol/L	18	13	90.0 (68.3–98.8)	87.6 (79.8–93.2)	58.1 (39.1–75.4)	97.9 (92.5–99.7)	7.3 (4.3–12.3)	0.1 (0–0.4)

*WHO* World Health Organization; *CI* confidence interval; *PPV* positive predictive value; *NPV* negative predictive value; *LR+* positive likelihood ratio; *LR–* negative likelihood ratio

### Daily changes in PVL and daily fluid intake in patients with severe and non-severe dengue

**Fig. 3:**
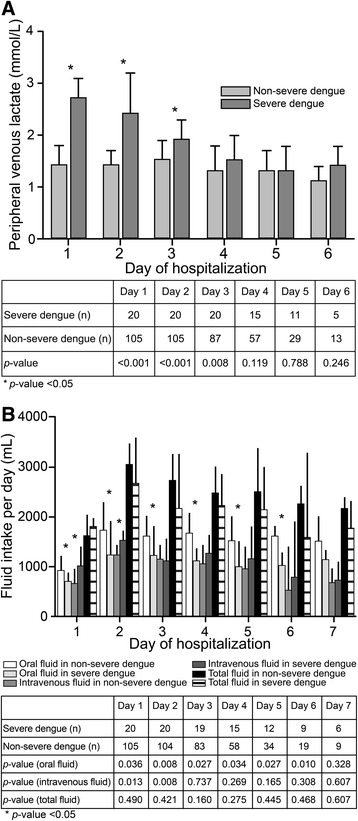
Changes in peripheral venous lactate and fluid intake among severe and non-severe dengue patients

We analyzed the daily changes in PVL levels during hospitalization among patients with severe and non-severe dengue. Patients with severe dengue had significantly higher PVL levels (compared to patients with non-severe dengue) at admission through day 3 of hospitalization. However, they exhibited a decrease in median PVL levels to <2.0 mmol/L on days 3 and thereafter (Fig. 3a). To determine the amount of fluid intake during hospitalization, we evaluated daily oral and intravenous fluid intake to determine the total fluid received among patients with severe and non-severe dengue. The amount of fluid intake was significantly lower among patients with severe dengue than those with non-severe dengue during day 1 through day 6 of hospitalization. In addition, the amount of intravenous fluid administered was significantly higher among patients with severe dengue than among those with non-severe dengue during day 1 and day 2 of hospitalization. However, the total fluid received was similar in both groups (Fig. 3b). Regarding the type of intravenous fluid, all patients with non-severe dengue received 0.9 % sodium chloride with or without dextrose as fluid therapy. Of 20 patients with severe dengue, 17 (85.0 %) patients received 0.9 % sodium chloride with or without dextrose and 3 (15.0 %) patients received Ringer’s acetate with or without dextrose as initial fluid resuscitation. However, 5 (25.0 %) patients received dextran and 4 (20.0 %) patients received 5 % albumin for additional fluid resuscitation.

## Discussion

A patient’s lactate level is a biomarker for the severity of systemic hypoperfusion independent of organ failure and shock [20]. In addition, elevated arterial or central venous lactate can predict in-hospital mortality in many conditions and diseases, including infection, sepsis, liver disease, trauma, and cardiac arrest [14]. Moreover, our previous studies revealed that hospitalized adults with dengue who had PVL levels ≥2.0 mmol/L at admission were at risk for the development of severe dengue (odd ratio, 7.340) [22]. Nevertheless, the utility of lactate levels for identifying severe dengue in adults has not been verified thus far, and we hypothesized that elevated lactate levels might be a diagnostic biomarker for patients with dengue and tissue hypoperfusion before they develop clinical symptoms of severe dengue.

Among the 125 hospitalized individuals with confirmed dengue viral infection, 105 (84.0 %) patients had non-severe dengue and 20 (16.0 %) patients had severe dengue. Interestingly, this incidence of severe dengue was lower than the incidence found in our previous retrospective study (27.9 %) [6]. This discrepancy may be due to the close observation and management of patients who participated in this prospective study. Our results also revealed that severe plasma leakage (75.0 %) was the most common complication among patients with severe dengue, which validates findings from a previous study [28]. Our analyses also revealed that most WSs (and the number of WSs) provided low specificities and sensitivities for identifying severe dengue. The one exception was the presence of clinical fluid accumulation (sensitivity: 75.0 %, specificity: 90.5 %, PPV: 60.0 %, NPV: 95.0 %, LR+: 7.9, and LR–: 0.3); previous studies have reported similar findings [8, 9].

When we evaluated the diagnostic value of PVL levels at admission, we found that the optimal PVL cutoff value of 2.5 mmol/L yielded a sensitivity of 65.0 %, specificity of 96.2 %, PPV of 76.5 %, NPV of 93.5 %, LR+ of 17.1, and LR– of 0.4. Similarly, a previous systematic review of lactate levels among patients with suspected infection revealed that patients with lactate levels of 2.0–3.9 mmol/L had an increased risk of progressive organ dysfunction and in-hospital mortality, including patients without hypotension [29, 30]. The combined biomarker of clinical fluid accumulation and/or PVL level of ≥2.5 mmol/L provided the maximum diagnostic accuracy (sensitivity: 90.0 %, specificity: 87.6 %, PPV: 58.1 %, NPV: 97.9 %, LR+: 7.3, and LR–: 0.1) for identifying severe dengue. These findings might be due to plasma leakage resulting from increased microvascular permeability that then leads to elevated hematocrit levels and/or clinical fluid accumulation; this may subsequently cause impaired microvascular perfusion, resulting in elevated lactate levels and eventually shock with organ dysfunction [31, 32]. Therefore, the combined biomarker of clinical fluid accumulation and/or PVL levels ≥2.5 mmol/L could provide the maximum diagnostic accuracy for identifying severe dengue.

Regarding fluid therapy for patients with dengue included in this study, the type and amount of fluid therapy were adjusted on the basis of the WHO South East Asia Regional Organization (SEARO) guidelines [2]. The majority of patients received 0.9 % sodium chloride with or without dextrose as initial fluid therapy. In patients with severe dengue, colloids including dextran and 5 % albumin were used as additional fluid resuscitation. Patients with severe dengue received a significantly lower amount of oral fluid intake per day during day 1 and day 6 of hospitalization, but the amount of intravenous fluid administered per day was significantly higher during day 1 and day 2 of hospitalization. However, the total fluid intake per day was similar in both groups. After fluid therapy, the PVL levels decreased, but the PVL levels were significantly higher among patients with severe dengue than among those with non-severe dengue during day 1 and day 3 of hospitalization.

Our study was designed as a prospective observational study, which could minimize the risk of missing data and reduce bias. Blood samples were regularly collected under blinded conditions for treating physicians and investigators in order to demonstrate, without bias, the dynamic changes of PVL throughout the febrile phase as well as the critical and recovery periods. However, this study had some limitations. First, we recruited only patients with dengue at a single center for tropical diseases in Thailand, which is likely not representative of the global population of patients with dengue. Second, we enrolled only patients who were hospitalized with dengue. Third, patients were admitted to the hospital at different times after fever onset, but all patients with dengue were admitted during the febrile phase.

## Conclusions

The presence of clinical fluid accumulation and/or PVL of ≥2.5 mmol/L could be used as a diagnostic biomarker for severe dengue among patients hospitalized with dengue. This biomarker might facilitate early recognition and timely treatment of patients with severe dengue, which can reduce dengue-related mortality, hospital burden, and unnecessary hospitalizations.

## Abbreviations

ALT: Alanine aminotransferase
AST: Aspartate aminotransferase
AUROC: Area under the receiver operating characteristic
CI: Confidence interval
ELISA: Enzyme-linked immunosorbent assays
IgM: Immunoglobulin M
IgG: Immunoglobulin G
IQR: Inter-quartile range
LR–: Negative likelihood ratio
LR+: positive likelihood ratio
NPV: Negative predictive value
PPV: Positive predictive value
PVL: Peripheral venous lactate
ROC: Receiver operating characteristic
RT-PCR: Reverse-transcriptase polymerase chain reaction
STARD: Standards for reporting of diagnostic accuracy
WBC: White blood cell count
WSs: Warning signs
WHO: World Health Organization
